# Antigen-Specific vs. Neutralizing Antibodies Against Conditioned Media of Patients With *Clostridioides difficile* Infection: A Prospective Exploratory Study

**DOI:** 10.3389/fmicb.2022.859037

**Published:** 2022-02-24

**Authors:** Sophie Roth, Philipp Jung, James Boone, Alexander Mellmann, Anna Nimmesgern, Sören L. Becker, Fabian K. Berger, Lutz von Müller

**Affiliations:** ^1^Institute of Medical Microbiology and Hygiene, Saarland University, Homburg, Germany; ^2^TechLab Research and Development, Blacksburg, VA, United States; ^3^Institute for Hygiene, University Hospital Münster, Münster, Germany; ^4^German National Reference Center for Clostridioides difficile, Homburg, Germany; ^5^Institute for Laboratory Medicine, Microbiology and Hygiene, Coesfeld, Germany

**Keywords:** *Clostridium difficile*, humoral immune response, antibody, vaccine, treatment

## Abstract

The immunological response against *Clostridioides difficile (C. difficile)* is crucial for an improved understanding of disease mechanisms and the development of novel therapeutic strategies. From April 2014 to February 2015, adult patients with *C. difficile* infection (CDI) were recruited, and the clinical course and treatment response were carefully monitored. On day 1, 3, and 6 after diagnosis, patient plasma samples were screened for anti-GDH (glutamate dehydrogenase), anti-TcdA, anti-TcdB, and anti-CWP84 (cell-wall protein 84) antibodies by ELISA. Additionally, neutralization assays of toxins from conditioned media of clinical isolates (RT010, RT014, and RT027) were performed. Most patients with CDI (*n* = 46) had antibodies against GDH (85%) and CWP84 (61%), but only few had antibodies against TcdA (11%) and TcdB (28%). We found patients with neutralizing antibodies against *C. difficile* toxins (conditioned media) produced by RT027 (26%). A subgroup of these samples could neutralize both toxins from RT027 and RT014 [11%, (5/46)]; however, no single sample neutralized only RT014. Overall, neutralizing antibody titers were low (≤1:16). In a one week follow-up of acute infection, we never observed an early booster effect with seroconversion or antibody increases, irrespective of disease severity. No correlation was found between the presence of antigen-specific (ELISA) or neutralizing antibodies and the clinical course of disease. Anti-TcdB but not anti-TcdA antibodies correlated with the occurrence of neutralizing antibodies. In conclusion, natural antibody titers against *C. difficile* toxins were absent or low and were not associated with disease severity. The correlation between the anti-TcdB with toxin neutralization confirms the importance of TcdB for virulence of CDI. Alternative sensitization strategies, e.g., through vaccine development, are required to overcome the regular low-titer antibody production following natural intestinal *C. difficile* exposure.

## Introduction

*Clostridioides difficile* is a Gram positive, anaerobic, spore-forming bacterium, and the major cause of infectious nosocomial diarrhea. Toxin production is considered as the main pathogenic factor for disease development. Toxigenic *C. difficile* strains harbor two toxins (TcdA and TcdB). In addition, some “hypervirulent” strains, such as ribotype 027 (RT027), express the binary toxin (CDT) of still unresolved clinical importance. RT027 and other “hypervirulent” strains exhibit higher levels of toxin production causing more severe course of disease (Warny et al., 2005).

The spectrum of CDI-related symptoms ranges from mild to severe courses of diseases (Napolitano and Edmiston, 2017; McDonald et al., 2018) with recurrence occurring in ≤30% of cases (McDonald et al., 2018). Risk factors for disease development and recurrence include among others antibiotic treatment, advanced age (≥ 65 years), or immunosuppression (Loo et al., 2011; Cózar-Llistó et al., 2016; Napolitano and Edmiston, 2017). However, intestinal dysbiosis with limited neutralization capacity of toxins seems to be crucial for disease development (Kyne et al., 2001; McDonald et al., 2018).

Oral antibiotic therapy of *C. difficile* is currently considered the mainstay of treatment for CDI (McDonald et al., 2018). However, these therapies pose the risk of recurrence.

While usage of toxin absorbing substances (Tolevamer) was effective in animal models but not in humans (Johnson et al., 2014), there are few studies suggesting a correlation between CDI-associated antibodies and a reduced risk of recurrence (Gupta et al., 2016; Kelly et al., 2019). Hence, promising approaches for new preventive therapies, such as monoclonal antibodies (Johnson and Gerding, 2019) or vaccines (Bézay et al., 2016; Kitchin et al., 2020), are currently developed. The first monoclonal antibody (Bezlotoxumab) was approved in 2017 for prevention of recurrences by neutralizing *C. difficile* toxin B (TcdB; Johnson and Gerding, 2019).

For further development of therapeutic strategies, the natural immune response seems to be of utmost importance. Likewise, antibodies against toxins and other *C. difficile*-specific targets may elaborate protective effects of humoral immune response. The common *C. difficile* antigen [glutamate dehydrogenase (GDH)] and cell wall-associated proteins (surface-layer proteins, SLP) may be involved. CWP84 is a paralogue of SLPs responsible for cleavage of SLP precursors into high molecular weight (HMW) and low molecular weight (LMW) subunits (de la Riva et al., 2011). Although cited very often in major textbooks, the association between occurrence of antibodies and clinical outcome is still debated (Gilbert et al., 2021), as data on antibody formation and their ability to neutralize toxins in the acute phase are limited.

Therefore, the aim of this prospective single-center study was to investigate the presence and dynamics of natural antibody response during the acute phase of CDI (anti-TcdA, anti-TcdB, anti-GDH, anti-CWP84, and neutralizing antibodies) and to assess the corresponding clinical course of disease.

## Materials and Methods

### Study Cohort

This prospective study was conducted at a Tertiary Care University Medical Center in Germany from April 2014 to February 2015 and included adult CDI patients (≥18 years). From each patient, informed consent was gathered. At study entry, patients’ characteristics were recorded (antibiotic use, hospitalization in the last three months, living in nursing homes, and immunocompromising disease or immunosuppressive therapy). The clinical course and antibiotic therapy were monitored during one week clinical follow-up. Moreover, all patients were contacted by phone after six to 12 weeks following CDI diagnosis to assess the recurrence rate.

The severity of disease was classified in three groups: transient (self-limiting diarrhea), mild, and severe (including fever ≥38.5°C, leukocytosis ≥15,000/μl), or increased creatinine (≥1.5 mg/dl).

### Diagnostic Sampling and Microbiological Analysis

*C. difficile* was initially detected in unformed stool specimens by application of a rapid test (C. DIFF QUIK CHEK COMPLETE^®^; TECHLAB, Inc., Blacksburg, United States), which was followed by anaerobic toxigenic culture with ribotyping of isolates as described previously (Berger et al., 2018). In short, *C. difficile* isolates were cultured under anaerobic conditions on selective media (CLO agar, bioMérieux, Marcy-l’Étoile, France). *C. difficile* isolates were identified using MALDI TOF (Bruker Daltonics, Billerica, United States) and underwent ribotyping and toxin gene detection in accordance to a harmonized protocol with the ribotyping primers described by Bidet et al. (1999). Symptomatic patients with detectable toxins in stool samples were included. EDTA plasma was obtained during consecutive follow-up (day 1, 3, and 6).

### Toxin Neutralization Test

#### *C. difficile* Toxins in Conditioned Media

For toxin neutralization test (NT), natural toxins were produced *in vitro* by *C. difficile* strains RT014 and RT027 from clinical isolates verified by ribotyping (institutional strain collection). These two strains were selected due their epidemiologic importance in Germany. As a negative control, conditioned media of the non-toxigenic RT010 (institutional strain collection) were used. Dynamics of toxin production with *C. difficile* growth kinetics were evaluated before for optimized collection of conditioned media with highest toxin levels.

Conditioned media (BHI liquid media, BD, United States) with high titers of natural *C. difficile* toxins (RT014, RT027) were harvested 96 h after anaerobic culture. After centrifugation, supernatants were sterile filtered (pore size 0.2 μm), aliquoted, and stored (−70°C) for subsequent investigations. Cytotoxic effects of conditioned media were confirmed by co-incubation with cell cultures (Vero B4 cells) and a colorimetric vitality assay (MTT, Sigma, M-2128, United States).

#### Neutralization Assay

Due to genotypic and also structural differences of *C. difficile* toxins related to various RTs (Rupnik, 2008; Hernandez et al., 2015; Fühner et al., 2018), toxins derived from two different pathogenic isolates were used (RT014, RT027).

Aliquots of patient plasma were stored (−20°C) and tested in small batches for the presence of neutralizing antibodies. Vero B4 cells were used as targets (10^4^ cells in 100 μl RPMI per well; Nunc^™^ MicroWell^™^ 96-Well, Nunclon Delta-Treated, Thermo Fisher Scientific, United States). Serial dilutions of samples (1:1,000 dilution with RPMI 1650) were incubated overnight (37°C) together with conditioned media (25 μl each) and confluent Vero B4 cells. Cytopathic effects of Vero B4 cells were assessed by inverted microscopy (ZEISS Axiovert 10, Zeiss, Germany). For each experiment, positive controls (RPMI instead of toxin for 100% vitality) and negative controls (RPMI instead of plasma for 100% cytotoxity) were included.

#### ELISA (Antibodies to Specific *C. difficile* Antigens)

Stored patient plasma (−20°C) was tested for the presence of specific antibodies to four different *C. difficile* antigens by ELISA. The following purified antigens were used: Recombinant glutamate dehydrogenase (rGDH), recombinant cell-wall protein 84 (rCWP84), native purified Toxin A (TcdA), and Toxin B (TcdB). Purified antigens were immobilized on a 96-well ELISA plate and then blocked using a bovine serum albumin (BSA). An anti-human Ig-HRP-conjugate and a 1-step TMB substrate (OD 450/620 nm) was used. Patient samples were tested at an initial dilution of 1:1,000. If necessary (OD >1.000), samples were diluted further. OD results were corrected accordingly by subtracting background signals (wells without antigens and blocked with BSA). A positive control consisting of pooled serum that reacted with all four antigens was done with every run of test samples.

#### Statistical Analysis

Fisher’s exact test was used to assess statistically significant differences. Values of *p*<0.05 were considered statistically significant. The software GraphPad Prism version 6.04 (GraphPad Software, San Diego, California, United States) was used.

## Results

### Growth Kinetics and Toxin Production *in vitro*

**Figure 1:**
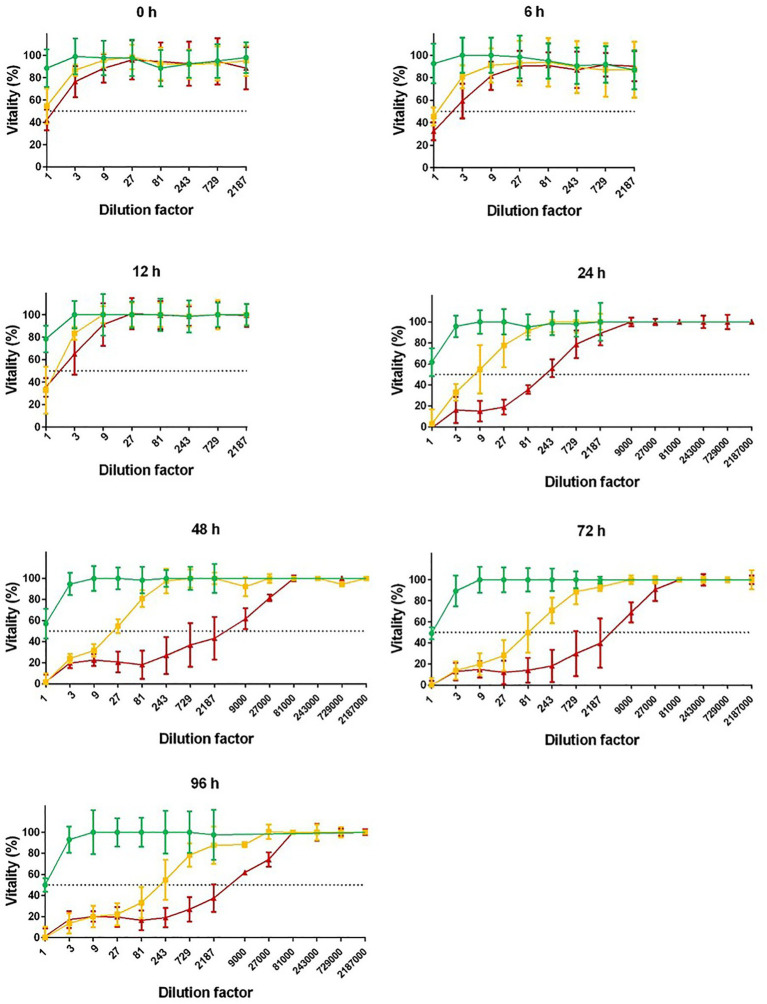
Cytotoxicity of different *Clostridioides difficile* ribotypes. Conditioned media (BHI) of RT010 (green), RT014 (yellow), and RT027 (red) *C. difficile* strains were harvested after 0, 6, 12, 24, 48, 72, and 96 h anaerobic culture. Cytotoxicity was tested in serial dilution with Vero cells in 96-well cell culture wells. Cytotoxicity 50% (CD50%) titers were calculated (dotted lines). Non-toxigenic RT010 served as negative control. Unspecific toxicity of undiluted media (titer <1:9) was not included.

**Figure 2:**
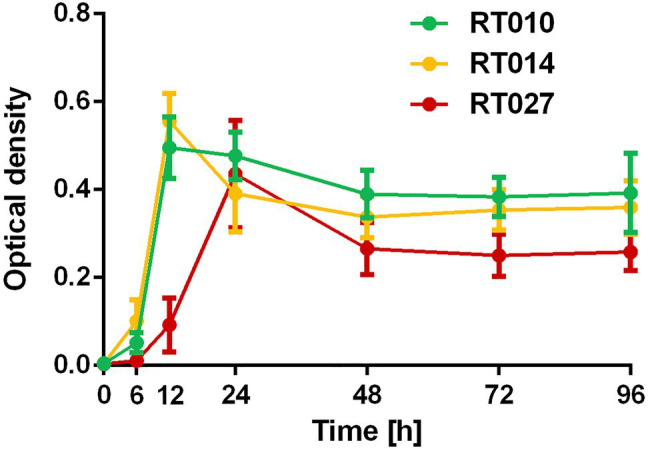
Growth curve of *C. difficile* RT010 (green), RT014 (yellow), and RT027 (red). Conditioned media (BHI) were harvested after 0, 6, 12, 24, 48, 72, and 96 h anaerobic culture and optical density were measured.

The growth of the investigated *C. difficile* strain RT027 in liquid cultures resulted in higher toxin production as compared to the strain RT014. The cytotoxic titer was 1:9,000 for RT014 and 1:27,000 for RT027 (96 h anaerobic culture in BHI liquid media). We confirmed that conditioned media of the strain RT010 were not toxic (negative control; Figure 1). Enhanced toxin production of RT027 strain occurred despite delayed growth kinetics as compared to RT014 and RT010 strains (Figure 2).

Quantitative cytotoxic effects of conditioned media following anaerobic culture with BHI media were determined by analyzing cytotoxicity dose 50% (CD50%). Cytotoxicity of concentrated conditioned media (titer <1:9) was excluded due to unspecific toxic effects of used media. Cytotoxicity of conditioned media (CD50%) was always higher for RT027 as compared to RT014: Cytotoxicity dose 50% (CD50%) of RT027 conditioned media was 24-fold higher to RT014 after 24 h. After 48 h, the difference was 170-fold higher, after 72 h 48-fold higher, and after 96 h (time of harvesting media supernatants) 25-fold higher.

For RT027, a plateau with highest cytotoxicity was achieved after 48 h (maximum CD50%: titer >1:3,000), while for RT014, the toxin production was lower and increased until end of cultivation time (maximum CD50%: titer 1:195; Figure 1).

### Patient Characteristics

The patients’ characteristics are shown in Supplementary Table 1. A total of 46 adult patients (median age 68, range 47–96) with newly diagnosed CDI were prospectively included. Informed consent was obtained. A total of 70% (32/46) of patients were diagnosed with a first episode of CDI and 30% (14/46) had a recurrence.

On average, diarrhea lasted 3.6 days (±2.1). A total of 20% (6/31) reported recurrent symptoms at follow-up which was performed after 6–12 weeks. Unfortunately, 15 patients were lost to follow-up. The results of ribotyping are included in Supplementary Figure 1.

### *C. difficile*-Specific Antibodies

A low abundance of anti-TcdA (11%, 5/46) and anti-TcdB (28%, 13/46) was detected in contrast to higher abundance of antibodies to both toxin-unrelated targets: Anti-GDH was positive in 85% (39/46) and anti-CWP84 in 61% (28/46) of patients.

Neutralizing antibodies against RT027 toxins were detected in only 26% of patients (12/46). Five of these 12 patients (corresponding to 11% of all patients) could also neutralize RT014 toxins. Of note, no single sample could neutralize RT014 in the absence of RT027 toxin neutralization.

Overall, the presence of *C. difficile* antibodies was not associated with typical risk factors.

*C. difficile* seroprevalence of voluntary healthy controls (*n* = 10, median age 29, range 21–62) was analyzed and again, the positive rate of anti-*C. difficile* antibodies (ELISA and NT) was low: 30% anti-TcdB, 30% anti-GDH, and 50% anti-CWP84. Anti-TcdA was not detected in healthy controls while 30% had neutralizing antibodies against both RT027 and RT014 toxins. Neutralization of RT027 or RT014 toxins alone was not observed in healthy controls (data not shown).

### Direct Comparison of Anti-TcdA, Anti-TcdB, and Neutralizing Antibodies

**Figure 3:**
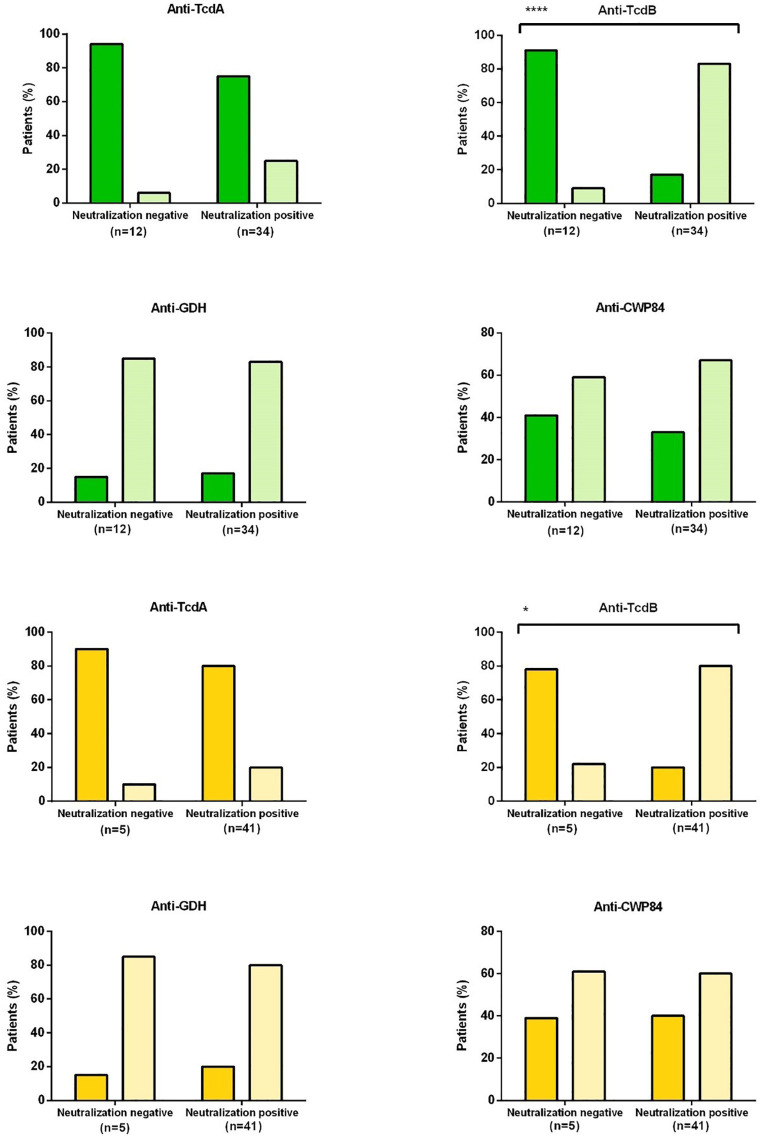
Correlation between enzyme immunoassay (EIA) and toxin neutralization assay (NT). The bars represent the percentage of patients with neutralizing or non-neutralizing antibodies (x-axis) and positive (light color) or negative (dark color) ELISA antibodies. Green bars refer to studies with conditioned medium of RT 027 and yellow to studies with conditioned medium RT014. Dark green or dark yellow: ELISA antibodies negative; light green or light yellow: ELISA antibodies positive TcdA, Toxin A; TcdB, Toxin B; GDH, glutamate dehydrogenase; CWP84, cell-wall protein 84. A significant correlation was found between anti-TcdB with neutralization of RT027 toxins (^****^p = 0,0001) and RT014 toxins (^*^p = 0,018).

The presence of anti-TcdB antibodies (ELISA) correlated significantly with neutralizing antibodies to RT027 toxins (*p* < 0.0001) and RT014 toxins (*p* = 0,018), respectively (Figure 3; Supplementary Table 2). No correlation was found between the presence of neutralizing antibodies and antibodies against TcdA, GDH, and CWP84. Furthermore, there was also no association between the specificity for RT027 and RT014 toxin neutralization and the presence of a specific corresponding ribotype.

### *C. difficile* Antibodies and Disease

Surprisingly, the presence of antigen specific (ELISA) and neutralizing (NT) antibodies was not associated with symptoms, severity of disease, therapy, treatment response, and recurrences (Supplementary Table 1). No significant correlations were observed between the detection of specific antibodies and patient characteristics in terms of risk factors or underlying disease. There was also no correlation between detection of antibodies and primary vs. recurrent diseases.

### Dynamics of Antibody Response After CDI

**Figure 4:**
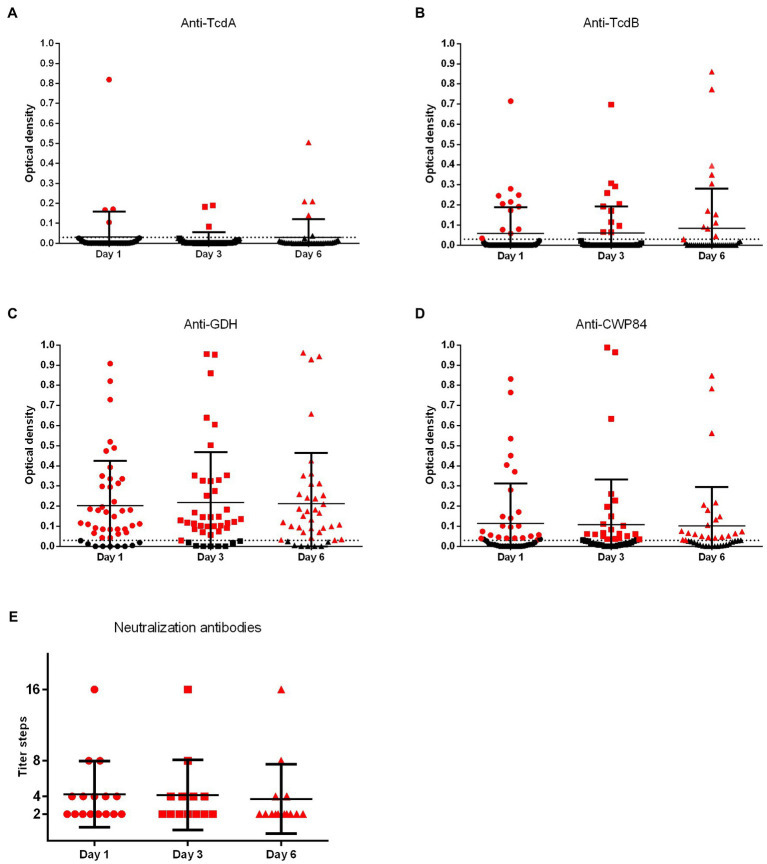
Quantitative values of *C. difficile*-specific antibodies. Consecutive plasma samples were investigated 1, 3, and 6 days after diagnosis of CDI. Anti-TcdA, anti-TcdB, anti-CWP84, and anti-GDH were investigated by ELISA (TcdA, Toxin A; TcdB, Toxin B; GDH, glutamate dehydrogenase; CWP84, cell-wall protein 84). OD values of ≥0.03 were considered positive for ELISA (dotted line). Neutralizing antibodies to RT014 and/or RT027 were investigated with Vero B cells and neutralization titers were determined by serial dilutions.

During the investigated early phase of infection (1, 3, and 6 days after diagnosis), there was no increase of antigen-specific (ELISA) or neutralizing (NT) antibodies (Figures 3, 4). An early booster effect was ruled out. Quantitative *C. difficile* antibody detection remained unchanged irrespective of primary infections vs. recurrence or severity of disease (Supplementary Table 1). Later time points were not available due to shortage of hospital stay.

## Discussion

Humoral immunity is crucial for treatment and prevention of a variety of toxin-related diseases, including CDI. Despite the frequently cited importance of antibodies in CDI, knowledge about natural antibody response is still limited (Viscidi et al., 1983; Kyne et al., 2001; Wullt et al., 2012; Gupta et al., 2016; von Eichel-Streiber et al., 2016; Kelly et al., 2019; Gilbert et al., 2021).

Only a low prevalence of anti-TcdA and of anti-TcdB antibodies were detected in our CDI patients, which is in line with previous studies (Kelly et al., 2019), and rates were only slightly higher in studies with prolonged follow-up (Bacon and Fekety, 1994; von Eichel-Streiber et al., 2016).

During the first week after CDI diagnosis, antibody titers remained stable and booster effects were not found, similarly to Wullt et al. (2012) which showed no significant anti-TcdA increase and only a moderate increase for anti-TcdB during prolonged follow-up. Kyne et al. (2001) could demonstrate a slight increase of anti-TcdA-IgG for patients with first episodes of CDI, but also stable antibody titers in patients with recurrence. However, in the previous aforementioned study, the dynamics of antibody response were marginal and gradual differences compared to our study might be related to a prolonged observation period, which might have captured a delayed antibody response.

Antibody detection during the first days of disease will very likely represent the preformed B-cell memory rather than a primary immune response, as antibody prevalence in studies with healthy control groups (Bacon and Fekety, 1994; von Eichel-Streiber et al., 2016) were comparable to those observed in the present study. Small differences could be related to methods but also to the fact that their patient cohort consisted of younger patients with no underlying diseases in contrast to our older patients with a history of numerous hospitalizations associated with an increased risk of *C. difficile* colonization.

Few studies focused on antibodies to toxin-unrelated antigens, such as GDH or CWP84. Overall, a study of Péchiné et al. showed a similar prevalence and stable titers of anti-CWP84 (Péchiné et al., 2005).

To our best knowledge, this is the first study investigating the prevalence and course of anti-GDH antibodies. In fact, seroprevalence of anti-GDH was high compared to other *C. difficile*-specific antibodies indicating prior *C. difficile* contact. Surprisingly, only 30% of our small healthy control group were anti-GDH positive. Increased anti-GDH seroprevalence of CDI patients could be explained by extended exposure of chronically ill patients to *C. difficile* contaminants in the healthcare system. Furthermore, antibodies could be elicited by other clostridial species (e.g., *C. clostridioforme*) due to cross-reactivity. Similar to other *C. difficile* antibodies, anti-GDH titers remained stable without significant booster effects.

GDH is the common *C. difficile* antigen secreted with high abundance following infection with *C. difficile* strains, irrespective of being toxigenic or non-toxigenic (Girinathan et al., 2014) and almost all individuals have repeated and regular *C. difficile* exposure since early infancy (Sánchez-Hurtado et al., 2008; Kelly and Kyne, 2011). It is tempting to speculate that anti-GDH might be used as a diagnostic parameter to estimate the overall *C. difficile* seroprevalence in the population. However, we found an anti-GDH prevalence ≤85% in CDI patients as well as heathy controls, which suggests that this parameter is not well suited to be used as a general indicator of past *C. difficile* history.

In the present study, 26% of CDI patients had neutralizing antibodies against RT027 toxins and only 11% against both toxins (RT027 and RT014). Seroprevalence was also low for neutralizing antibodies of heathy controls (30%). Before, Viscidi et al. (1983) described also low seroprevalence of neutralizing antibodies against TcdB (22%) as well as TcdA (9%). Furthermore, in recent vaccination studies, the seroprevalence and also quantitative titers of neutralizing antibodies were low in unvaccinated controls (Kitchin et al., 2020). Low or absent neutralizing antibody titers at onset of disease in combination with missing dynamics of antibody response in the early phase of CDI may explain why natural antibodies to *C. difficile* and its toxins did not influence the clinical CDI course (von Eichel-Streiber et al., 2016). No correlation could be found between the presence of neutralizing antibodies and disease severity, which is consistent with previous studies (von Eichel-Streiber et al., 2016; Kelly et al., 2019).

In our study, the presence of anti-TcdB was correlated with the presence of neutralizing antibodies against RT014 and RT027 toxins. Viscidi et al. (1983) suggested that anti-TcdB and anti-TcdA correlated with neutralization. In the present study, a correlation with anti-TcdA was ruled out. Discrepant results might have occurred prior due to cross-reactivity of diagnostic antigen preparations while highly purified toxins were now available for the present study.

High relapse rates were also found among anti-TcdB positive (38%) and NT positive patients (25%). One other recent study also failed to demonstrate an association between the presence of anti-TcdB and the risk of recurrence (Gilbert et al., 2021).

However, anti-TcdB titers can be obtained by vaccines which is different to regular antibody production after natural intestinal contact (Kitchin et al., 2020) and are required for preventing CDI recurrence (Gupta et al., 2016; Kelly et al., 2019) demonstrated by successful passive immunization with Bezlotoxumab and active toxoid vaccination (Kitchin et al., 2020).

While TcdB is accepted to be the major toxin associated with CDI, the clinical role of TcdA is still a matter of debate. In this study, anti-TcdB but not anti-TcdA was correlated to toxin neutralization confirms the fact that TcdB is the predominant toxin related to cytopathic effects and presumably also to toxin-related disease.

Interestingly, neutralization capacity of RT014 and RT027 toxins was different for a number of patients. Twelve patients had neutralizing antibodies to RT027 toxins and five of them to RT014 toxins also. No single sample could neutralize RT014 alone.

Thus, neutralizing antibodies were always active against RT027 toxins while antibodies directed against RT014 toxins only were not present in our study. This main finding confirms the assumption that toxigenic *C. difficile* strains express conserved but also strain-specific neutralizing TcdB epitopes (Supplementary Figure 2; Hernandez et al., 2015). Antibodies against conserved epitopes potentially bind and neutralize TcdB of different RTs, which was demonstrated here for a minority of patients with the capacity of RT014 and RT027 toxin neutralization. In contrast, antibodies against epitopes in variable regions would only be able to neutralize toxins of specific strains.

This single-center study has several limitations. This is a pilot study with a small cohort of patients and controls. A substantial loss of long-term follow-up is intrinsic to almost all studies with *C. difficile* dealing with an elderly multimorbid cohort. These limitations for statistical analysis may be overcome by larger, prospective multi-center studies. Early booster effects after CDI could be ruled out for all *C. difficile* antibodies investigated. However, we cannot exclude a delayed antibody response because samples after prolonged period of times were missing. This remains to be focused in future studies, especially to collect an antibody status before any recurrence. Furthermore, neutralizing antibodies should also be tested against conditioned media of other endemic and epidemic ribotypes (e.g., RT001) in comparison to distinguish strain-specific from broad-spectrum epitopes of antibodies.

## Conclusion

The natural *C. difficile* antibody response was low. Booster effects with seroconversion or increased antibody production were not evident within the first week after CDI. Presence of specific antibodies was not associated with patients’ characteristics, clinical symptoms, or disease severity. Anti-TcdB was directly correlated with toxin neutralization. Samples with broad-range and with strain-restricted neutralization capacity were found, which might be related to different neutralizing epitopes.

## Data Availability Statement

The original contributions presented in the study are included in the article/Supplementary Material, further inquiries can be directed to the corresponding author.

## Ethics Statement

The studies involving human participants were reviewed and approved by Saarland Medical Council No. 20/14. The patients/participants provided their written informed consent to participate in this study.

## Author Contributions

LvM: conceptualization and supervision. LvM, SR, AN, and JB: methodology. SR and JB: investigation. SR: data curation and writing-original draft preparation. LM, JB, SLB, FB, PJ, and AM: writing-review and editing. SR and PJ: visualization. All authors contributed to the article and approved the submitted version.

## Funding

The present work was supported by an unrestricted grant from TechLab, Inc., Blacksburg, Virginia and by the German National Reference Center for *Clostridioides (Clostridium)* difficile supported with an unrestricted grant from the Robert Koch Institute, Germany.

## Conflict of Interest

JB is employee at TechLab Research and Development, FB received consultant fees from MSD and Pfizer.

The remaining authors declare that the research was conducted in the absence of any commercial or financial relationships that could be construed as a potential conflict of interest.

## Publisher’s Note

All claims expressed in this article are solely those of the authors and do not necessarily represent those of their affiliated organizations, or those of the publisher, the editors and the reviewers. Any product that may be evaluated in this article, or claim that may be made by its manufacturer, is not guaranteed or endorsed by the publisher.
