# Supplementary material for: Antigen-Specific vs. Neutralizing Antibodies Against Conditioned Media of Patients With Clostridioides difficile Infection: A Prospective Exploratory Study
Source: Front Microbiol. 2022 Feb 24;13:859037. doi: 10.3389/fmicb.2022.859037 (PMC8908241; doi:10.3389/fmicb.2022.859037)
Supplement: Supplementary file 1 [file Table_1.DOCX]

**Supplementary Table 1:** Clinical characteristics of patients and corresponding antibodies.

|  | **Patients n (%)** | **anti RT027 toxin (NT)** | **anti RT 027 and 014 toxin (NT)** | **None (NT)** | **anti-TcdA (EIA)** | **anti-TcdB (EIA)** | **anti-GDH (EIA)** | **anti-CWP84 (EIA)** |
| --- | --- | --- | --- | --- | --- | --- | --- | --- |
| **Total** | 46/46 (100) | 12/46 (26) | 5/46 (11) | 34/46 (74) | 5/46 (11) | 3/46 (28) | 39/46 (85) | 28/46 (61) |
| **Patient details** |  |  |  |  |  |  |  |  |
| Female | 22/46 (48) | 6/12 (50) | 1/5 (20) | 16/34 (47) | 1/5 (20) | 6/13 (46) | 21/39 (54) | 12/28 (43) |
| Male | 24/46 (52) | 6/12 (50) | 4/5 (80) | 18/34 (53) | 4/5 (80) | 7/13 (54) | 18/39 (46) | 16/28 (57) |
| Age (years) | 69 (±11,3) | 68 (±13) | 68 (±13) | 67 (±13) | 67 (±13) | 67 (±12) | 69 (±11) | 70 (±12) |
| **Risk factors** |  |  |  |  |  |  |  |  |
| Previous antibiotic treatment | 34/45*(76) | 8/12 (67) | 3/5 (60) | 26/33* (79) | 2/5 (40) | 10/13 (77) | 30/39 (77) | 20/28 (71) |
| Hospitalization in the last 3 months | 30/45* (67) | 10/12 (83) | 4/5 (80) | 20/33* (61) | 4/5 (80) | 11/13 (85) | 26/39 (67) | 16/28 (57) |
| Nursing home resident | 4/45* (9) | 0/12 (0) | 0/5 (0) | 4/33* (12) | 0/5 (0) | 1/13 (8) | 4/39 (10) | 3/28 (11) |
| **Comorbidities** |  |  |  |  |  |  |  |  |
| Oncological disease | 22/45* (49) | 7/12 (58) | 4/5 (80) | 15/33* (45) | 1/5 (20) | 5/13 (38) | 17/39 (44) | 12/28 (43) |
| Hepatic insufficiency | 2/45* (4) | 2/12 (17) | 2/5 (40) | 0/33* (0) | 1/5 (20) | 2/13 (15) | 2/39 (5) | 2/28 (7) |
| Kidney failure | 13/45* (29) | 4/12 (33) | 1/5 (20) | 9/33* (27) | 2/5 (40) | 4/13 (31) | 11/39 (28) | 8/28 (29) |
| Chronic lung disease | 8/45* (18) | 2/12 (17) | 1/5 (20) | 6/33* (18) | 0/5 (0) | 2/13 (15) | 8/39 (21) | 4/28 (14) |
| Immunosuppression | 8/45* (18) | 2/12 (17) | 1/5 (20) | 6/33* (18) | 1/5 (20) | 2 (15) | 7/39 (18) | 4/28 (14) |
| **Symptoms** |  |  |  |  |  |  |  |  |
| Fever | 5/46 (12) | 2/12 (17) | 1 /5 (20) | 3/34 (10) | 0/5 (0) | 1/13 (8) | 3/39 (8) | 3/28 (12) |
| Diarrhoea | 3,6 d (± 2,1) | 3,7d (± 2,0) | 3,7d (± 2,0) | 3,6 d (± 2,1) | 3,6 d (± 2,1) | 3,7 (± 2,0) | 3,6 (± 2,1) | 3,6 (± 2,0) |
| **Severity of disease** |  |  |  |  |  |  |  |  |
| Transient | 4/46 (9) | 2/12 (17) | 1/5 (20) | 2/34 (6) | 0/5 (0) | 0/13 (0) | 2/39 (5) | 1/28 (4) |
| Mild | 1746 (37) | 3/12 (25) | 1/5 (20) | 14/34 (41) | 2/5 (40) | 4/13 (31) | 15/39 (38) | 13/28 (46) |
| Severe | 25/46 (54) | 7/12 (58) | 3/5 (60) | 18/34 (53) | 3/5 (60) | 9/13 (69) | 22/39 (56) | 14/28 (50) |
| **Therapy** |  |  |  |  |  |  |  |  |
| No therapy | 6/46 (13) | 3/12 (25) | 1/5 (20) | 3/34 (9) | 0/5 (0) | 1/13 (8) | 4/39 (10) | 3/28 (11) |
| Metronidazol | 32/46 (70) | 8/12 (67) | 4/5 (80) | 24/34 (71) | 4/5 (80) | 10/13 (77) | 28/39 (72) | 18/28 (64) |
| Vancomycin | 3/46 (7) | 1/12 (8) | 0/5 (0) | 2/34 (6) | 1/5 (20) | 2/13 (15) | 3/39 (8) | 3/28 (11) |
| Vancomycin and Metronidazol | 5/46 (11) | 0/12 (0) | 0/5 (0) | 5/34 (15) | 0/5 (0) | 0/13 (0) | 4/39 (10) | 4/28 (14) |
| **Therapy response** |  |  |  |  |  |  |  |  |
| ˂ 48h | 15/37* (41) | 3/9* (33) | 1/4* (25) | 12/28* (43) | 0/5 (0) | 5/12* (42) | 13/32*(41) | 9/23* (39) |
| ˂ 72h | 4/37* (11) | 0/9* (0) | 0/4* (0) | 4/28* (14) | 1/5 (20) | 0/12* (0) | 4/32* (13) | 2/23* (9) |
| ≥ 72h | 14/37* (38) | 4/9* (44) | 1* (25) | 10/28* (36) | 4/5 (80) | 5/12* (42) | 12/32* (38) | 10/23* (43) |
| No response | 4/37*(11) | 2/9* (22) | 2/4* (50) | 2/28* (7) | 0/5 (0) | 2/12* (17) | 3/32* (9) | 2/23* (9) |
| **Follow up** |  |  |  |  |  |  |  |  |
| Total | 31/31 (100) | 7/31 (23) | 2/31 (6) | 24/31 (77) | 3/31 (26) | 8/31 (26) | 25/31 (81) | 16/31 (52) |
| Recurrence | 6/31 (19) | 1/7 (14) | 0/2 (0) | 5/24 (21) | 1/3 (33) | 2/8 (25) | 5/25 (20) | 4/16 (25) |


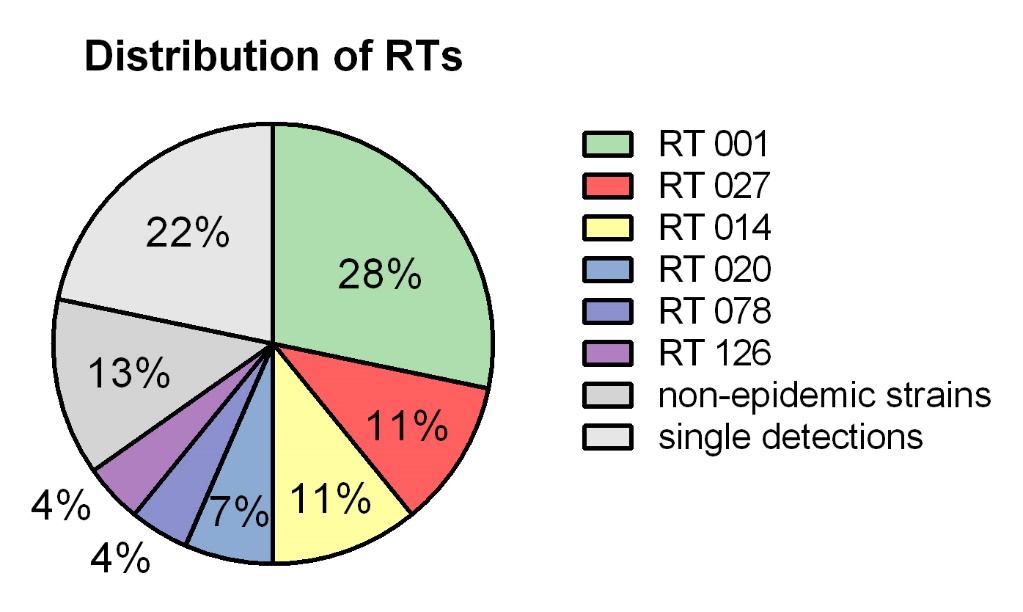


**Supplementary Figure 1**: PCR Ribotypes of 46 patients with CDI. RT001 (n=13), RT027 (n=5), RT014 (n=5), RT020 (n=3), RT078 (n=2) and RT126 (n=2). RT005, RT011, RT012, RT017, RT023, RT029, RT046, RT053, RT081 and RT159 were detected once. Few sporadic cases (n=6) without match to known ribotypes of the institutional database additional isolates were also found.

**Supplementary Table 2:** Direct comparison between anti-TcdA and anti-TcdB with toxin neutralization. A significant correlation was found between anti-TcdB and neutralization of RT027 toxins (***, p = 0,0001) and neutralization of RT014 toxins (*, p = 0,018)

|  | NT positive  (RT027) | NT negative  (RT027) | NT positive  (RT014) | NT negative  (RT014) |
| --- | --- | --- | --- | --- |
| Anti-TcdA positive (ELISA) | 3 | 2 | 1 | 4 |
| Anti-TcdA negative (ELISA) | 9 | 32 | 4 | 37 |
| Anti-TcdB positive (ELISA) | 10 *** | 3 | 4 * | 9 |
| Anti-TcdB negative (ELISA) | 2 | 31 | 1 | 32 |

**Supplementary Figure 2:** Sequence alignment for TcdB of RT014 and RT027. Differences with amino acid (AA) exchanges were shown. Also, neutralizing epitope 1 (yellow) and epitope 2 (pink) for Bezlotoxumab were shown (Hernandez et al 2015).

RT027_2700649_tcdB MSLVNRKQLEKMANVRFRVQEDEYVAILDALEEYHNMSENTVVEKYLKLKDINSLTDIYI 60

RT014_2700952_tcdB MSLVNRKQLEKMANVRFRTQEDEYVAILDALEEYHNMSENTVVEKYLKLKDINSLTDIYI 60

******************.*****************************************

RT027_2700649_tcdB DTYKKSGRNKALKKFKEYLVTEVLELKNNNLTPVEKNLHFVWIGGQINDTAINYINQWKD 120

RT014_2700952_tcdB DTYKKSGRNKALKKFKEYLVTEVLELKNNNLTPVEKNLHFVWIGGQINDTAINYINQWKD 120

************************************************************

RT027_2700649_tcdB VNSDYNVNVFYDSNAFLINTLKKTIVESATNDTLESFRENLNDPRFDYNKFYRKRMEIIY 180

RT014_2700952_tcdB VNSDYNVNVFYDSNAFLINTLKKTVVESAINDTLESFRENLNDPRFDYNKFFRKRMEIIY 180

************************:**** *********************:********

RT027_2700649_tcdB DKQKNFINYYKTQREENPDLIIDDIVKIYLSNEYSKDIDELNSYIEESLNKVTENSGNDV 240

RT014_2700952_tcdB DKQKNFINYYKAQREENPELIIDDIVKTYLSNEYSKEIDELNTYIEESLNKITQNSGNDV 240

***********:******:******** ********:*****:********:*:******

RT027_2700649_tcdB RNFEEFKGGESFKLYEQELVERWNLAAASDILRISALKEVGGVYLDVDMLPGIQPDLFES 300

RT014_2700952_tcdB RNFEEFKNGESFNLYEQELVERWNLAAASDILRISALKEIGGMYLDVDMLPGIQPDLFES 300

*******.****:**************************:**:*****************

RT027_2700649_tcdB IEKPSSVTVDFWEMVKLEAIMKYKEYIPGYTSEHFDMLDEEVQSSFESVLASKSDKSEIF 360

RT014_2700952_tcdB IEKPSSVTVDFWEMTKLEAIMKYKEYIPEYTSEHFDMLDEEVQSSFESVLASKSDKSEIF 360

**************.************* *******************************

RT027_2700649_tcdB SSLGDMEASPLEVKIAFNSKGIINQGLISVKDSYCSNLIVKQIENRYKILNNSLNPAISE 420

RT014_2700952_tcdB SSLGDMEASPLEVKIAFNSKGIINQGLISVKDSYCSNLIVKQIENRYKILNNSLNPAISE 420

************************************************************

RT027_2700649_tcdB DNDFNTTTNAFIDSIMAEANADNGRFMMELGKYLRVGFFPDVKTTINLSGPEAYAAAYQD 480

RT014_2700952_tcdB DNDFNTTTNTFIDSIMAEANADNGRFMMELGKYLRVGFFPDVKTTINLSGPEAYAAAYQD 480

*********:**************************************************

RT027_2700649_tcdB LLMFKEGSMNIHLIEADLRNFEISKTNISQSTEQEMASLWSFDDARAKAQFEEYKKNYFE 540

RT014_2700952_tcdB LLMFKEGSMNIHLIEADLRNFEISKTNISQSTEQEMASLWSFDDARAKAQFEEYKRNYFE 540

*******************************************************:****

RT027_2700649_tcdB GSLGEDDNLDFSQNTVVDKEYLLEKISSLARSSERGYIHYIVQLQGDKISYEAACNLFAK 600

RT014_2700952_tcdB GSLGEDDNLDFSQNIVVDKEYLLEKISSLARSSERGYIHYIVQLQGDKISYEAACNLFAK 600

************** *********************************************

RT027_2700649_tcdB TPYDSVLFQKNIEDSEIAYYYNPGDGEIQEIDKYKIPSIISDRPKIKLTFIGHGKDEFNT 660

RT014_2700952_tcdB TPYDSVLFQKNIEDSEIAYYYNPGDGEIQEIDKYKIPSIISDRPKIKLTFIGHGKDEFNT 660

************************************************************

RT027_2700649_tcdB DIFAGLDVDSLSTEIETAIDLAKEDISPKSIEINLLGCNMFSYSVNVEETYPGKLLLRVK 720

RT014_2700952_tcdB DIFAGFDVDSLSTEIEAAIDLAKEDISPKSIEINLLGCNMFSYSINVEETYPGKLLLKVK 720

*****:**********:***************************:************:**

RT027_2700649_tcdB DKVSELMPSISQDSIIVSANQYEVRINSEGRRELLDHSGEWINKEESIIKDISSKEYISF 780

RT014_2700952_tcdB DKISELMPSISQDSIIVSANQYEVRINSEGRRELLDHSGEWINKEESIIKDISSKEYISF 780

**:*********************************************************

RT027_2700649_tcdB NPKENKIIVKSKNLPELSTLLQEIRNNSNSSDIELEEKVMLAECEINVISNIDTQVVEGR 840

RT014_2700952_tcdB NPKENKITVKSKNLPELSTLLQEIRNNSNSSDIELEEKVMLTECEINVISNIDTQIVEER 840

******* *********************************:*************:** *

RT027_2700649_tcdB IEEAKSLTSDSINYIKNEFKLIESISDALYDLKQQNELEESHFISFEDILETDEGFSIRF 900

RT014_2700952_tcdB IEEAKNLTSDSINYIKDEFKLIESISDALCDLKQQNELEDSHFISFEDISETDEGFSIRF 900

*****.**********:************ *********:********* **********

RT027_2700649_tcdB IDKETGESIFVETEKAIFSEYANHITEEISKIKGTIFDTVNGKLVKKVNLDATHEVNTLN 960

RT014_2700952_tcdB INKETGESIFVETEKTIFSEYANHITEEISKIKGTIFDTVNGKLVKKVNLDTTHEVNTLN 960

*:*************:***********************************:********

RT027_2700649_tcdB AAFFIQSLIEYNSSKESLSNLSVAMKVQVYAQLFSTGLNTITDAAKVVELVSTALDETID 1020

RT014_2700952_tcdB AAFFIQSLIEYNSSKESLSNLSVAMKVQVYAQLFSTGLNTITDAAKVVELVSTALDETID 1020

************************************************************

RT027_2700649_tcdB LLPTLSEGLPVIATIIDGVSLGAAIKELSETSDPLLRQEIEAKIGIMAVNLTAATTAIIT 1080

RT014_2700952_tcdB LLPTLSEGLPIIATIIDGVSLGAAIKELSETSDPLLRQEIEAKIGIMAVNLTTATTAIIT 1080

**********:*****************************************:*******

RT027_2700649_tcdB SSLGIASGFSILLVPLAGISAGIPSLVNNELILRDKATKVVDYFSHISLAESEGAFTSLD 1140

RT014_2700952_tcdB SSLGVASGFSILLVPLAGISAGIPSLVNNELVLRDKATKVVDYFKHVSLVETEGVFTLLD 1140

****:**************************:************.*:**.*:**.** **

RT027_2700649_tcdB DKIMMPQDDLVISEIDFNNNSITLGKCEIWRMEGGSGHTVTDDIDHFFSAPSITYREPHL 1200

RT014_2700952_tcdB DKIMMPQDDLVISEIDFNNNSIVLGKCEIWRMEGGSGHTVTDDIDHFFSAPSITYREPHL 1200

**********************.*************************************

RT027_2700649_tcdB SIYDVLEVQKEELDLSKDLMVLPNAPNRVFAWETGWTPGLRSLENDGTKLLDRIRDNYEG 1260

RT014_2700952_tcdB SIYDVLEVQKEELDLSKDLMVLPNAPNRVFAWETGWTPGLRSLENDGTKLLDRIRDNYEG 1260

************************************************************

RT027_2700649_tcdB EFYWRYFAFIADALITTLKPRYEDTNIRINLDSNTRSFIVPVITTEYIREKLSYSFYGSG 1320

RT014_2700952_tcdB EFYWRYFAFIADALITTLKPRYEDTNIRINLDSNTRSFIVPIITTEYIREKLSYSFYGSG 1320

*****************************************:******************

RT027_2700649_tcdB GTYALSLSQYNMNINIELNENDTWVIDVDNVVRDVTIESDKIKKGDLIENILSKLSIEDN 1380

RT014_2700952_tcdB GTYALSLSQYNMGINIELSESDVWIIDVDNVVRDVTIESDKIKKGDLIEGILSTLSIEEN 1380

************.*****.*.*.*:************************.***.****:*

RT027_2700649_tcdB KIILDNHEINFSGTLNGGNGFVSLTFSILEGINAVIEVDLLSKSYKVLISGELKTLMANS 1440

RT014_2700952_tcdB KIILNSHEINFSGEVNGSNGFVSLTFSILEGINAIIEVDLLSKSYKLLISGELKILMLNS 1440

****:.******* :**.****************:***********:******* ** **

RT027_2700649_tcdB NSVQQKIDYIGLNSELQKNIPYSFMDDKGKENGFINCSTKEGLFVSELSDVVLISKVYMD 1500

RT014_2700952_tcdB NHIQQKIDYIGFNSELQKNIPYSFVDSEGKENGFINGSTKEGLFVSELPDVVLISKVYMD 1500

* :********:************:*.:******** *********** ***********

RT027_2700649_tcdB NSKPLFGYCSNDLKDVKVITKDDVIILTGYYLKDDIKISLSFTIQDENTIKLNGVYLDEN 1560

RT014_2700952_tcdB DSKPSFGYYSNNLKDVKVITKDNVNILTGYYLKDDIKISLSLTLQDEKTIKLNSVHLDES 1560

:*** *** **:**********:* ****************:*:***:*****.*:***.

RT027_2700649_tcdB GVAEILKFMNKKGSTNTSDSLMSFLESMNIKSIFINSLQSNTKLILDTNFIISGTTSIGQ 1620

RT014_2700952_tcdB GVAEILKFMNRKGNTNTSDSLMSFLESMNIKSIFVNFLQSNIKFILDANFIISGTTSIGQ 1620

**********:**.********************:* **** *:***:************

RT027_2700649_tcdB FEFICDKDNNIQPYFIKFNTLETKYTLYVGNRQNMIVEPNYDLDDSGDISSTVINFSQKY 1680

RT014_2700952_tcdB FEFICDENDNIQPYFIKFNTLETNYTLYVGNRQNMIVEPNYDLDDSGDISSTVINFSQKY 1680

******:::**************:************************************

RT027_2700649_tcdB LYGIDSCVNKVIISPNIYTDEINITPIYEANNTYPEVIVLDTNYISEKINININDLSIRY 1740

RT014_2700952_tcdB LYGIDSCVNKVVISPNIYTDEINITPVYETNNTYPEVIVLDANYINEKINVNINDLSIRY 1740

***********:**************:**:***********:***.****:*********

RT027_2700649_tcdB VWSNDGSDFILMSTDEENKVSQVKIRFTNVFKGNTISDKISFNFSDKQDVSINKVISTFT 1800

RT014_2700952_tcdB VWSNDGNDFILMSTSEENKVSQVKIRFVNVFKDKTLANKLSFNFSDKQDVPVSEIILSFT 1800

******.*******.************.****.:*:::*:********** :.::* :**

RT027_2700649_tcdB PSYYVEGLLNYDLGLISLYNEKFYINNFGMMVSGLVYINDSLYYFKPPIKNLITGFTTIG 1860

RT014_2700952_tcdB PSYYEDGLIGYDLGLVSLYNEKFYINNFGMMVSGLIYINDSLYYFKPPVNNLITGFVTVG 1860

**** :**:.*****:*******************:************::******.*:*

RT027_2700649_tcdB DDKYYFNPDNGGAASVGETIIDGKNYYFSQNGVLQTGVFSTEDGFKYFAPADTLDENLEG 1920

RT014_2700952_tcdB DDKYYFNPINGGAASIGETIIDDKNYYFNQSGVLQTGVFSTEDGFKYFAPANTLDENLEG 1920

******** ******:******.*****.*.********************:********

RT027_2700649_tcdB EAIDFTGKLTIDENVYYFGDNYRAAIEWQTLDDEVYYFSTDTGRAFKGLNQIGDDKFYFN 1980

RT014_2700952_tcdB EAIDFTGKLIIDENIYYFDDNYRGAVEWKELDGEMHYFSPETGKAFKGLNQIGDYKYYFN 1980

********* ****:***.****.*:**: **.*::*** :**:********** *:***

RT027_2700649_tcdB SDGIMQKGFVNINDKTFYFDDSGVMKSGYTEIDGKYFYFAENGEMQIGVFNTADGFKYFA 2040

RT014_2700952_tcdB SDGVMQKGFVSINDNKHYFDDSGVMKVGYTEIDGKHFYFAENGEMQIGVFNTEDGFKYFA 2040

***:******.***:..********* ********:**************** *******

RT027_2700649_tcdB HHDEDLGNEEGEALSYSGILNFNNKIYYFDDSFTAVVGWKDLEDGSKYYFDEDTAEAYIG 2100

RT014_2700952_tcdB HHNEDLGNEEGEEISYSGILNFNNKIYYFDDSFTAVVGWKDLEDGSKYYFDEDTAEAYIG 2100

**:********* :**********************************************

RT027_2700649_tcdB ISIINDGKYYFNDSGIMQIGFVTINNEVFYFSDSGIVESGMQNIDDNYFYIDENGLVQIG 2160

RT014_2700952_tcdB LSLINDGQYYFNDDGIMQVGFVTINDKVFYFSDSGIIESGVQNIDDNYFYIDDNGIVQIG 2160

:*:****:*****.****:******::*********:***:***********:**:****

RT027_2700649_tcdB VFDTSDGYKYFAPANTVNDNIYGQAVEYSGLVRVGEDVYYFGETYTIETGWIYDMENESD 2220

RT014_2700952_tcdB VFDTSDGYKYFAPANTVNDNIYGQAVEYSGLVRVGEDVYYFGETYTIETGWIYDMENESD 2220

************************************************************

RT027_2700649_tcdB KYYFDPETKKAYKGINVIDDIKYYFDENGIMRTGLITFEDNHYYFNEDGIMQYGYLNIED 2280

RT014_2700952_tcdB KYYFNPETKKACKGINLIDDIKYYFDEKGIMRTGLISFENNNYYFNENGEMQFGYINIED 2280

****:****** ****:**********:********:**:*:*****:* **:**:****

RT027_2700649_tcdB KTFYFSEDGIMQIGVFNTPDGFKYFAHQNTLDENFEGESINYTGWLDLDEKRYYFTDEYI 2340

RT014_2700952_tcdB KMFYFGEDGVMQIGVFNTPDGFKYFAHQNTLDENFEGESINYTGWLDLDEKRYYFTDEYI 2340

* ***.***:**************************************************

RT027_2700649_tcdB AATGSVIIDGEEYYFDPDTAQLVISE* 2366

RT014_2700952_tcdB AATGSVIIDGEEYYFDPDTAQLVISE* 2366

***************************
